# Influence of dextran deswelling on graft preparation and clinical outcomes in DMEK

**DOI:** 10.1007/s10561-026-10247-x

**Published:** 2026-08-10

**Authors:** N. Kalantari, G. Lang, G. Geerling, S. B. Zwingelberg

**Affiliations:** 1 Department of Ophthalmology, Faculty of Medicine and University Hospital of Düsseldorf, University of Düsseldorf, Moorenstr. 5, 40225 Düsseldorf, Germany; 2 Department of Functional Materials in Medicine and Dentistry, University Hospital Würzburg, Würzburg, Germany; 3 Department of Ophthalmology, Faculty of Medicine and University Hospital of Cologne, University of Cologne, Cologne, Germany

**Keywords:** Descemet membrane endothelial keratoplasty (DMEK), Organ culture, Dextran

## Abstract

To evaluate the influence of dextran-containing versus dextran-free organ culture media on graft preparation characteristics and postoperative outcomes following Descemet Membrane Endothelial Keratoplasty (DMEK). In this single-center retrospective comparative study, 31 organ-cultured corneoscleral donor rims obtained from the Eye Bank of the University Hospital of Cologne between 2018 and 2022 were analyzed. Donor age ranged from 52 to 74 years. Donor tissues were allocated to either dextran-containing organ culture medium (group A, n = 16) or dextran-free medium (group B, n = 15) according to logistical and surgical scheduling considerations. Intraoperative graft preparation characteristics were assessed using a standardized scoring system evaluating Descemet membrane splitting and stripping behavior, graft fragility, and central and peripheral attachment tendencies. Postoperative outcome parameters included Best Spectacle-Corrected Visual Acuity (BSCVA), central and peripheral corneal thickness (CCT and PCT), endothelial cell count (ECC), and re-bubbling rate over a follow-up period of 12 months after DMEK. All 31 DM-endothelium grafts were successfully prepared and transplanted for DMEK. No significant differences were observed between swollen and dextran-deswollen grafts regarding intraoperative preparation characteristics, including stripping behavior (*p* = 0.722), graft fragility (*p* = 0.433), peripheral attachment tendency (*p* = 1.000), and central tearing tendency (*p* = 0.484). Descemet membrane splitting behavior was also comparable between groups. At 12 months postoperatively, no significant differences were detected between groups regarding CCT (group A: 517.67 ± 15.10 µm vs. group B: 530.10 ± 24.92 µm; *p* = 0.189) or re-bubbling rate (*p* = 0.704). A trend toward lower PCT was observed in group B (group A: 604.00 ± 21.67 µm vs. group B: 579.50 ± 36.12 µm; *p* = 0.063), accompanied by a trend toward higher ECC in group B (group A: 1387 ± 131 cells/mm^3^ vs. group B: 1692 ± 242 cells/mm^3^; *p* = 0.066). BSCVA at 12 months was significantly better in the dextran-free group (group A: 0.08 ± 0.38 logMAR vs. group B: 0.03 ± 0.13 logMAR; *p* = 0.032). Swollen and dextran-deswollen donor grafts demonstrated comparable intraoperative preparation characteristics and similar postoperative outcomes following DMEK. The slightly improved visual outcomes observed in the dextran-free group suggest that dextran supplementation during organ culture may not be necessary for DMEK graft preparation. Avoiding dextran may further reduce time and costs in corneal eye banking. Larger prospective studies are warranted to confirm these findings.

## Introduction

Due to unsurpassed results with minimal risk of graft rejection, Descemet membrane endothelial keratoplasty (DMEK) has successively become the principal grafting technique for the treatment of corneal endothelial diseases such as Fuchs endothelial corneal dystrophy (FECD) or bullous keratopathy (BK) (Melles 2006; Hos et al. 2019; Flockerzi et al. 2018; Matthaei et al. 2019).

Before transplantation donor corneas are typically stored in an organ culture system at 32 °C for up to 35 days in Europe, while the prevailing method in the United States is cold storage at 4 °C for a maximum period of 14 days (Association 2006, 2007; Thuret et al. 2005a; Want et al. 1983). Massive stromal swelling occurs under organ culturing conditions, which is why the graft is transferred into a deswelling medium before penetrating keratoplasty (PK) or deep anterior lamellar keratoplasty (DALK) (Borderie et al. 1997; Thuret et al. 2005b). Deswelling is achieved by applying a hyperosmolar medium usually containing dextran at least 24 h before transplantation (Zhao et al. 2008). However, prolonged culturing in dextran-containing medium is associated with a loss of corneal endothelial cells (Pels and Schuchard 1984; Salla et al. 1995). Since Descemet membrane (DM) does not swell, transfer of the donor cornea into a deswelling medium is not needed for DMEK. Eye banks have now started to prepare DMEK precut grafts without prior deswelling step.

Nevertheless, the influence of dextran on the graft preparation for DMEK procedure and their outcomes has not still been sufficiently examined. The aim of this study was to investigate the influence and presence of dextran with regard to tissue preparation for the DMEK procedure and the subsequent postoperative outcome of swollen versus dextran-swollen DM endothelial grafts in those DMEK.

## Methods

### Inclusion and exclusion criteria

This single-center retrospective comparative observational study evaluated 31 organ-cultured donor corneas obtained between 2018 and 2022 from the Eye Bank of the University Hospital of Cologne. Prior to DMEK preparation, corneas were cultured either in dextran-containing medium (group A) or dextran-free medium (group B). Allocation to the respective culture protocol was based on logistical and surgical scheduling considerations. Specifically, donor corneas intended as potential backup tissue for penetrating keratoplasty (PK) or deep anterior lamellar keratoplasty (DALK) were transferred into dextran-containing deswelling medium before surgery. This strategy minimized systematic allocation bias between the study groups and enabled greater flexibility in graft reassignment if a PK or DALK procedure required urgent replacement tissue.

To improve comparability of graft preparation conditions, donor age was restricted to 52–74 years, as a wider age distribution may increase variability in graft preparability. Corneas were cultured for a maximum of 30 days in a medium containing Aqua bidest, minimal essential medium (MEM) with Earle’s salts, penicillin, streptomycin, amphotericin B, HEPES buffer, and fetal calf serum. During cultivation, regular medium exchanges and endothelial cell counts were performed. A final medium exchange was carried out at least 24 h before the planned surgery using either medium containing 6% dextran T-500 (group A) or dextran-free medium (group B). The decision to place a cornea in deswelling medium before preparation depended exclusively on logistical considerations related to the surgical schedule. Corneoscleral donor tissue obtained from external eye banks was excluded from the analysis.

Additional exclusion criteria for the outcome analysis included extracorneal visual acuity–limiting conditions such as age-related macular degeneration, diabetic retinopathy, macular edema, advanced glaucoma, and amblyopia, as well as previous ocular surgeries including vitrectomy, glaucoma-filtering procedures, or prior corneal surgery. Eyes with a follow-up period of less than 12 months were also excluded.

The study adhered to the tenets of the Declaration of Helsinki and complied with all applicable German federal and state regulations. Data collection was approved by the local Institutional Review Board.

### Clinical information and outcome parameters

Demographic data including age and gender of the donors and recipient were recorded. The following intraoperative preparation characteristics were evaluated by the surgeons using a standardized scoring system (s. Table 1): Descemet’s membrane splitting (1) and the fragility (2) of DM during preparation, peripheral attachment (3), central tearing (4) as well as the severity of stripping (5). Moreover, graft preparation failure was noted in addition. The culture time was noted from all organ-cultured corneoscleral rims for DMEK. In addition, for simplification and better comparability four of the five criteria were used for a score to assess difficulty of DMEK graft preparation, based on the scorings system of Schrittenlocher et al. (2022). The score included as above described the (1) existence of central Descemet membrane attachments, (2) existence of peripheral Descemet membrane attachments in grafts harvested from pseudophakic eyes, (3) occurrence of Descemet membrane splitting and (4) fragility of the Descemet endothelium complex. The tissue was regarded as fragile if the tissue fell apart when touched by a forceps. If all four criteria were negative and answered with “no”, then the surgery was categorized as “easy preparation” (group 1).

**Table 1:** Overview of collected data inclusive demographics and the intraoperative preparation data of the grafts

	Group A n	Group A %	Group B n	Group B %	*p*-value
n_patients_	15		16		
Age ± SD [years]	63.0 ± 7.2		64.9 ± 6.4		0.448
Gender male donor	11	73.3	14	87.5	0.394
Gender female donor	4	26.7	2	12.5	0.394
Splitting	15	100	16	100	0.113
Yes	6	40.0	2	12.5	
No	9	60.0	14	87.5	
Fragility	15	100	16	100	0.433
Yes	12	80.0	10	62.5	
No	3	20.0	6	37.5	
Peripheral attachment	15	100	16	100	1.000
Yes	4	26.7	4	25.0	
No	11	73.3	12	75.0	
Central attachment	15	100	16	100	0.484
Yes	0	0.0	2	12.5	
No	15	100	14	87.5	
Score	15	100	16	100	0.542
Group I	3	20.0	6	37.5	
Group II	6	40.0	6	37.5	
Group III	6	40.0	4	25.0	
Stripping difficulties	15	100	16	100	0.720
Yes	8	53.3	10	62.5	
No	7	46.7	6	37.5	

If one criterion was answered with “yes” and the other three with “no”, then we assigned the surgery to group 2 (“difficult preparation”). If two or more criteria were answered with “yes”, the surgery was assigned to group 3 (“very difficult preparation”). Thus, it was possible to standardize and reliably record the graft preparability in group A and B.

The outcome parameters after DMEK were defined by using the best spectacle-corrected visual acuity (BSCVA), the central and peripheral corneal thickness (CCT and PCT), the endothelial cell count (ECC) and the re-bubbling rate. The follow-up period was a minimum of 12 months in all patients after DMEK procedure. Pre-operative data and 1–3, 6 and 12-months’ follow-up data were collected and evaluated.

### Surgical technique

DMEK graft preparation was performed directly before transplantation by experienced surgeons (Prof. Björn Bachmann and Prof. Claus Cursiefen) as previously described: (Matthaei et al. 2019; Schaub et al. 2020; Dapena et al. 2011; Kruse et al. 2014; Schrittenlocher et al. 2018). The preparation started after mounting the donor cornea on a suction block endothelial side up (Hanna trephination system; Moria SA, Antony, France). First, the peripheral DM was scored using either a forceps or a razor blade. After staining with trypan blue 0.06% solution (Vision blue; DORC, Zuidland, The Netherlands) for about 10 s careful circular lifting of the edge of the DM was carried out giving special attention to small peripheral radial tears which were immediately removed by pulling one side of DM towards the periphery. The incomplete stripping was performed by pulling DM with a forceps leaving a central portion of about 1 × 1 mm attached. After trephination with an 8 mm trephine three orientation marks were placed with a 1 mm trephine at the edge of the graft. Following a second staining step for about 10 s stripping was completed by pulling DM with a forceps. Before loading into the DMEK shooter system the graft was transferred to a Petri dish containing the culture medium in which the graft was delivered to the operation theater. Graft preparations were performed directly before transplantation by the surgeon.

### Statistical analyses

All statistical analyses were performed using SPSS software version 25.0 (IBM Corp., Armonk, NY, USA). Data were analyzed descriptively and are presented as mean ± standard deviation (SD) where appropriate. Normal distribution of continuous variables was assessed using the Kolmogorov–Smirnov and Shapiro–Wilk tests.

Best Spectacle-Corrected Visual Acuity (BSCVA) values were converted to logarithm of the minimum angle of resolution (logMAR) units for statistical evaluation. Differences in demographic variables, intraoperative graft preparation characteristics, and postoperative outcome parameters, including central corneal thickness (CCT), peripheral corneal thickness (PCT), endothelial cell count (ECC), BSCVA, and re-bubbling rate, were analyzed between groups.

Continuous variables were compared using Student’s t-test, while categorical variables were analyzed using the Pearson chi-square test. A *p*-value < 0.05 was considered statistically significant.

## Results

### Demographics

The study cohort consisted of 31 donor corneas provided by the eye bank of the University Hospital of Cologne either in dextran-containing medium (group A; n = 16) or medium without dextran (group B; n = 15). The donor age in group A was 63.0 ± 7.2 years and 65.4 ± 6.6 in group B, *p* = 0.448. In groups A and B there was a normal distribution of the donor sex without a significant difference in the female-to-male ratio (*p* = 0.394). The culture time in group A was 17.3 ± 4.5 days and 20.3 ± 5.8 in group B, *p* = 0.172. The age of recipient in group A was 71.5 ± 9.5 years and 70.8 ± 9.6 group B (*p* = 0.836). There was a normal distribution of the sex of recipient in groups A and B with no significant difference in the female-to-male ratio (*p* = 0.728).

### Intra-operative preparation of DM graft

In both groups, all 31 DMEK graft preparations were successfully performed. In general no significant difference in the intraoperative preparation conditions of deswelling (group A) or a non-deswelling group (group B) DM-endothelium grafts were observed (Table 1):

Regarding the splitting behavior, no difference was detected between group A (n = 16) and group B (n = 15) with *p* = 0.113. The fragility of the grafts was also not statistically significant (*p* = 0.433), as well as the peripheral scars indicating a pseudophakic donor (*p* = 1.000) and the tendency for central tearing (*p* = 0.484).

In respect to the stripping behavior, in group A (n = 15) n = 8 (53.3%) showed stripping difficulties, while in n = 7 (46.7%) a completely free of complications stripping was notable.

There was no significant difference overall compared to group B (n = 16, *p* = 0.722) with n = 10 (62.5%) showing a more difficult stripping behavior, compared to n = 6 (37.5%) having no stripping difficulties.

As a result for the standardized scoring system, a score of I in n = 3 (20.0%), a score of II in n = 6 (40.0%) and a score of III in n = 6 (40%) are obtained in group A. In comparison to group B, no significance was detectable: Group B showed a score of I in n = 6 (37.5%) (*p* = 0.444), a score of II in n = 6 (37.5%) (*p* = 1.000) and a score of III in n = 4 ( 25.0%) (*p* = 0.542) in respect to the preparation conditions.

### Post operative outcome of swollen versus dextran deswollen DM-endothelium grafts in DMEK

All 31 DMEK procedure in group A and B were successfully performed. No significant difference in the outcome of using deswelling (group A) or a non-deswelling group (group B) DM-endothelium grafts for DMEK could be detected in respect to CCT and ECC and the re-bubbling rate (Table 2):

**Table 2:** Overview of outcome data of DM endothelial grafts de-swollen with dextran (group A) compared with swollen grafts (group B) in DMEK and italicized percent decrease (including range) in ECC, highlighted with*

	Group A	Group B	*p*-value 12 months after DMEK
Central corneal thickness, CCT [µm]			
Pre-operative	664.00 ± 56.57	664.44 ± 111.72	
1–3 months	532.71 ± 35.41	534.92 ± 33.03	
6 months	515.13 ± 27.48	548.57 ± 36.67	
12 months	517.67 ± 15.10	530.10 ± 24.92	0.189
Peripheral corneal thickness, PCT [µm]			
Pre-operative	700.21 ± 77.33	720.56 ± 127.18	
1–3 months	615.43 ± 36.23	608.42 ± 44.63	
6 months	598.25 ± 30.76	598.86 ± 51.95	
12 months	604.00 ± 21.67	579.50 ± 36.12	0.063
Endothelial cell count, ECC [cells/mm^3^]			
Donor	2884 ± 139	3022 ± 284	
1–3 months	2092 ± 50 (27.47%, range 21.97–32.46%)*	2293 ± 201 (24.13%, range 8.95–36.73%)*	
6 months	No data available (No data available)	1960 ± 279 (35.15%, range 18.22–49.16%)*	
12 months	1387 ± 131 (51.91%, range 44.34–58.46%)*	1692 ± 242 (44.02%, range 29.37–56.15%)*	0.066
Best spectacle-corrected Visual acuity, BSCVA [logMAR]			
Pre-operative	0.38 ± 0.16	0.37 ± 0.21	
1–3 months	0.15 ± 0.21	0.15 ± 0.27	
6 months	0.14 ± 0.06	0.06 ± 0.12	
12 months	0.08 ± 0.38	0.03 ± 0.13	0.032

#### Central corneal thickness (CCT)

The CCT decreased in group A from 664.00 ± 56.57 µm before surgery to 515.13 ± 27.48 µm six months and to 517.67 ± 15.09 µm 12 months after DMEK compared to group B (664.44 ± 111.72 µm before surgery, 548.57 ± 36.67 µm six months and 530.10 ± 25.00 µm 12 months after DMEK). Twelve months after DMEK no significant difference was observed in group A versus group B, *p* = 0.189, compare Table 2.

#### Peripheral corneal thickness (PCT)

Twelve months after DMEK a statistic borderline difference was observed in group B (without dextran) regarding PCT with *p* = 0.063, but however with a high effect power of 0.678: The PCT decreased from 700.21 ± 77.33 µm before surgery to 598.25 ± 30.76 µm six months and to 604.00 ± 21.67 µm 12 months after surgery in group A compared to group B with 720.56 ± 127.18 µm before surgery to 598.86 ± 51.95 µm six months and to 579.50 ± 36.12 µm 12 months after DMEK procedure (compare Table 2).

#### Endothelial cell count (ECC)

The mean Donor age in group A was 63.0 ± 7.2 years (range 52–74years) and the donor endothelial cell count before surgery was 2884 ± 139 cells/mm^3^.

The mean Donor age in group B was 65.4 ± 6.6 years (range 57–74years) and the donor endothelial cell count before surgery was 3022.94 ± 284 cells/mm^3^.

ECC in group A after one to three months was 2092 ± 50 cells/mm^3^ and showed a continuous decrease with an ECC of 1387 ± 132 cells/mm^3^ and a percent decrease of 51.91% (range 44.34–58.46%) 12 months after surgery. In group B ECC was after one to three months 2293 ± 202 cells/mm^3^ with a continuous decrease in ECC of 1692 ± 242 cells/mm^3^ (44.02%, range 29.37–56,15%) 12 months after surgery. Twelve months after DMEK, a trend toward higher ECC was observed in group B (without dextran) (*p* = 0.066), with a greater effect size (− 0.446); see Table 2.

#### Best spectacle-corrected visual acuity (BSCVA)

The BSCVA increased in group A from 0.38 ± 0.16 before surgery to 0.14 ± 0.06 six months and to 0.08 ± 0.38 twelve months after DMEK compared to group B (0.37 ± 0.21 before surgery, 0.06 ± 0.12 six months and 0.03 ± 0.13 twelve months after DMEK). BSCVA was significant better in group B (without dextran) 12 months after DMEK with *p* = 0.032 (Table 2).

#### Re-bubbling rate

The re-bubbling rate in both groups was comparable with 25.0% (n = 4) in group A versus 33.3% (n = 5) in group B, (*p* = 0.704). All patients received SF-6 gas in a non-expanding concentration of 20% for anterior chamber tamponade. The maximum number of re-bubblings in each group was one**.**

## Discussion

To our knowledge, this is the first study evaluating both intraoperative graft preparation characteristics and 12-month postoperative outcomes of swollen versus dextran-deswollen grafts in DMEK.

The following major novel findings emerge from this study:

I. The use of a dextran-containing medium demonstrates no advantages in terms of DM- splitting, the fragility of DM during preparation, the central tearing, the peripheral attachment, the severity of stripping and the graft preparation failure.
II. CCT, PCT and ECC were comparable in both groups 12 months after DMEK by using or not using dextran for the grafts during the culture procedure.
III. The BSCVA was significantly better in the group B, in which no dextran was used.
IV. Avoiding the use of Dextran seems to be a promising approach to reduce costs and time, thus optimizing the organ culture process in corneal eye banking effectively and sustainably.

To balance the onset of modifications triggered by the culture medium during culturing process, facilitate intraoperative preparation as well as the suturing of the graft and accelerate postoperative visual recovery, the cornea must be first thinned before surgery through immersion in the same organ culture medium supplemented with a water-soluble macromolecule in form of dextran that produces colloid osmotic pressure to extract excess water accumulated in the stroma. This deswelling process should be reduced to the shortest time possible to obtain a graft thickness similar to that of the recipient and to ensure the highest possible number of endothelial cells. In several studies before it was observed, that at the last pre-operative medium change into the dextran-containing medium decreased the endothelial cell number significantly, compared to the grafts in which no pre-operative de-swelling took place 24 h before surgery (Yoeruek et al. 2013; Salla et al. 2019). This suggests that dextran exposure may contribute to endothelial cell loss during organ culture (Borderie et al. 1997; Thuret et al. 2005b; Yoeruek et al. 2013; Salla et al. 2019). Thuret et al. (2005b) observed a 15% cell loss after 2 days of deswelling in commercial media containing 5% dextran. An average of an 8.4% endothelial cell loss was reported after 1–4 days of deswelling in an organ culture medium supplemented with 5% dextran in the study of Borderie et al. (1997).

Bartz Schmidt et al. (Yoeruek et al. 2013) demonstrated that DM endothelial grafts for DMEK procedures can be surgically prepared from organically cultured corneal rims in a swollen and de-swollen tissue condition. Both separation methods appear to be equivalent regarding the preparation characteristics of the obtained DM. In our study, regardless of whether dextran was used for de-swelling in the organ culture, no difference could be observed in the intraoperative surgical preparation conditions regarding the stripping, splitting and fragility of the grafts, as well as the central and peripheral attachment. Also by using the standardized preparation score of Schrittenlocher et al. (2022), no preparation differences could be objectively determined in de-swollen and non-de-swollen grafts.

The trend toward a higher postoperative endothelial cell density observed in the dextran-free group is consistent with previous studies reporting reduced endothelial toxicity in the absence of dextran-containing deswelling media (Borderie et al. 1997; Thuret et al. 2005b; Salla et al. 2019). Although the underlying mechanisms remain speculative, the higher endothelial cell density may have contributed to the more pronounced reduction in peripheral corneal thickness and the significantly better visual acuity observed in this cohort. One possible explanation is more effective endothelial pump function, resulting in more homogeneous corneal deturgescence across both the central and peripheral cornea. Such a uniform reduction in corneal thickness may promote more regular posterior corneal remodeling and reduce higher-order optical aberrations, and thereby facilitate superior visual rehabilitation.

An important aspect highlighted by recent eye-bank studies concerns the structural changes that develop during prolonged storage in dextran-free organ culture media. Parekh et al. demonstrated that prolonged storage without dextran results in pronounced stromal edema and Descemet membrane folds, whereas transfer into dextran-containing medium for 24–48 h before graft preparation effectively restores stromal dehydration, reduces Descemet membrane folds and is associated with lower endothelial cell mortality and fewer uncovered endothelial areas following graft preparation (Parekh et al. 2016, 2017). These findings suggest that transient mechanical stress induced by Descemet membrane folds may affect endothelial viability during stripping, even when postoperative endothelial cell density is ultimately preserved. However, the clinical relevance of these transient alterations remains to be established and requires confirmation in prospective studies integrating both standardized endothelial viability assessment and long-term clinical outcomes.

Conversely, more recent clinical studies have demonstrated that dextran-free preservation of pre-stripped or preloaded DMEK grafts can achieve excellent clinical outcomes following transplantation. Wallace et al. recently reported favorable multicenter clinical outcomes using dextran-free preservation media for preloaded endothelium-in grafts, whereas Abdin et al. similarly found no clinical advantage of dextran-containing media for pre-stripped DMEK tissue preservation (Wallace et al. 2026; Abdin et al. 2018). Our findings are in good agreement with these clinical observations, as we observed comparable intraoperative graft preparation characteristics, similar postoperative corneal thickness, endothelial cell density and rebubbling rates over 12 months despite omission of dextran during the final storage period.

Nevertheless, our study did not specifically evaluate endothelial viability immediately after graft preparation. We did not perform quantitative vital dye analysis, assessment of Trypan Blue-positive endothelial defects, or measurements of uncovered endothelial areas after stripping. During routine graft preparation, no consistent macroscopic differences in Trypan Blue staining patterns were observed between groups that interfered with transplantation. However, subtle alterations in endothelial viability associated with Descemet membrane folding cannot be excluded and may not necessarily be reflected by postoperative endothelial cell density measurements alone. Consequently, it remains possible that transient endothelial injury occurring during graft preparation was underestimated in the present study.

Recent advances in automated endothelial viability analysis may provide an opportunity to address this limitation. In particular, the recently validated deep learning-assisted platform developed by Airaldi et al. enables objective quantification of total endothelial cell viability immediately before transplantation and may represent a valuable adjunct for future eye-bank quality assessment (Airaldi et al. 2025).

Combining standardized viability assays, quantitative Trypan Blue analysis and artificial intelligence-assisted endothelial evaluation with long-term clinical follow-up will help clarify whether transient viability changes observed during graft preparation translate into clinically relevant long-term outcomes.

Taken together, our findings indicate that omission of dextran during the final storage period did not adversely affect intraoperative graft preparation or postoperative clinical outcomes within the limitations of this study. Additional limitations should nevertheless be acknowledged. Graft preparation was evaluated using a standardized surgeon-based scoring system, which inevitably contains a subjective component despite being performed by highly experienced DMEK surgeons. Furthermore, this retrospective non-paired study was not designed to detect subtle differences in endothelial viability immediately after stripping. Larger prospective eye-bank studies incorporating objective endothelial viability measurements are therefore warranted.

Avoiding dextran may additionally reduce processing time, tissue handling, and overall eye-bank costs while simplifying the organ culture workflow. However, these potential logistical advantages should be interpreted alongside the currently available experimental evidence and confirmed in prospective multicenter studies integrating both immediate endothelial viability analyses and long-term clinical outcomes.

## Conclusions

In this retrospective comparative study, donor corneas preserved with or without dextran supplementation during the final deswelling period demonstrated comparable intraoperative graft preparation characteristics and similar postoperative clinical outcomes following DMEK. Omission of dextran during the final deswelling period did not adversely affect graft preparation, postoperative corneal thickness, endothelial cell density, or rebubbling rates over 12 months. Although a trend toward higher postoperative endothelial cell density and significantly better visual acuity was observed in the dextran-free group, these findings should be interpreted with caution given the retrospective design and limited sample size. Avoiding dextran may simplify eye-bank processing while reducing tissue handling, processing time, and associated costs. However, larger prospective multicenter studies incorporating standardized endothelial viability assessment are required before dextran-free storage protocols can be recommended for routine clinical practice.

## Data Availability

The datasets generated and/or analyzed during the current study are available from the corresponding author upon reasonable request.
